# Healing and Hope for Forced Migrants in Norwich: The SHIFA Clinic for Asylum Seekers and Refugees

**DOI:** 10.1192/bjo.2024.376

**Published:** 2024-08-01

**Authors:** Yasir Hameed, Hannah Fox, Izobel Clegg, Eirini Charamiroupa, Meghana Rayala

**Affiliations:** 1 Norfolk and Suffolk NHS Foundation Trust, Norwich, United Kingdom; 2 Norfolk and Norwich University Hospital, Norwich, United Kingdom

## Abstract

**Aims:**

The poster will discuss our Quality Improvement project around improving access to mental health assessments for asylum seekers and refugees and why this model of care proved to be useful in reducing barriers to accessing specialist mental health services for these patients.

The clinic was launched as part of the Advancing Mental Health Equality (AMHE) Collaborative, which is a 3-year programme run by the National Collaborating Centre for Mental Health at the Royal College of Psychiatrists, and Norfolk and Suffolk NHS Foundation Trust (NSFT) was one of the organisations that signed up to this initiative.

**Methods:**

The clinic was named SHIFA, which stands for (Supporting Holistic and Integrated Forced Migrants Assessments). SHIFA means ‘Healing’ in Arabic, Turkish, Urdu, Kurdish and Pashtu (languages spoken by many patients accessing this clinic) and the name was selected in collaboration with staff and service users.

What are the objectives of the SHIFA clinic?

- To offer a trauma-informed approach to the assessment of forced migrants. This is an essential objective of the SHIFA clinic as the trauma-informed approaches are guided by trust, rapport and offering person-centred care.
- Reduce barriers to mental health care for people seeking asylum, refugees and people forced to migrate.
- Work collaboratively across the systems to bridge the gap among different services to improve the person-centred and continuity of care and avoid the re-traumatising effect of re-telling their stories.
- Co-production and putting the voice of our service users and carers at the heart of everything we do.
- Share learning and embed inclusive practice within the mental health services in NSFT and other organisations.

**Results:**

We will provide data on patients seen in the clinic, the diagnosis and treatment. We will also give examples of the feedback we received from professionals and service users and why this model successfully provided collaborative and joint working opportunities with various services working with these patients.

**Conclusion:**

The QI project represented in this clinic has provided a local solution to meeting the needs of forced migrants in our community, reducing mental health inequalities. It ran without funding initially and was successful in receiving funding due to the clear difference it made in providing good quality care for these patients.

Similar projects can be easily implemented in various mental health trusts.

